# Plant-soil interactions and C:N:P stoichiometric homeostasis of plant organs in riparian plantation

**DOI:** 10.3389/fpls.2022.979023

**Published:** 2022-08-01

**Authors:** Dongdong Ding, Muhammad Arif, Minghui Liu, Jiajia Li, Xin Hu, Qianwen Geng, Fan Yin, Changxiao Li

**Affiliations:** ^1^Key Laboratory of Eco-Environments in the Three Gorges Reservoir Region (Ministry of Education), Chongqing Key Laboratory of Plant Resource Conservation and Germplasm Innovation, College of Life Sciences, Southwest University, Chongqing, China; ^2^Biological Science Research Center, Academy for Advanced Interdisciplinary Studies, Southwest University, Chongqing, China

**Keywords:** Three Gorges Reservoir, Yangtze River, riparian zone, woody plants, plant growth, submergence

## Abstract

Carbon (C), nitrogen (N), and phosphorus (P) stoichiometric ratios give valuable insight into ecosystem function. The purpose of the present study is to probe into the C, N, and P stoichiometric characteristics in various organs and their relationships with soil factors of the dominant deciduous conifer plant species (*Taxodium ascendens* and *Taxodium distichum*) during afforestation in the riparian zone of Three Gorges Reservoir. The results showed only a small change in the concentration of C in different plant organs and soils. *T. ascendens* contained mean N and P concentrations of 7.63 and 1.54 g/kg in fine roots, 5.10 and 0.56 g/kg in stems, and 15.48 and 2.30 g/kg in leaves, respectively. Whereas *T. distichum* had a mean N and P concentration of 7.08 and 1.37 g/kg in fine roots, 4.84 and 0.59 g/kg in stems, and 16.89 and 2.23 g/kg in leaves. The N:P ratios in all organs were below 14, indicating that N may have inhibited tree growth. The fine roots P and N:P of *T. distichum* were weak plasticity and weak homeostasis, and those of *T. ascendens* were plasticity and weak plasticity. Their stems and leaves adhere to strict homeostasis. N concentrations were significantly positively related to P concentrations in every tissue (except the stems of *T. ascendens*), and C concentrations were significantly positively associated with P concentrations in the stems and leaves of *T. ascendens* and *T. distichum* (*p* < 0.05). Likewise, soil P and fine root P were positively associated (*p* < 0.01). This study contributes to the understanding of deciduous conifer plant stoichiometry. It demonstrates N, P, and N:P stoichiometric homeostasis in *T. ascendens* and *T. distichum*, which can withstand flooding and are suitable for vegetation restoration in the hydro-fluctuation zone.

## Introduction

Riparian areas play an important role in material circulation, energy flow, and ecosystem function and maintenance as a link between the aquatic and terrestrial ecosystems (Zaharescu et al., 2017; Yuan et al., 2021; Arif et al., 2022a). In addition to providing wildlife habitat, buffer zones allow terrestrial and aquatic organisms to move freely along the river systems to avoid the formation of isolated communities (Modrak et al., 2017; Zhu et al., 2019; Zheng et al., 2021a). Plants in the riparian zone provide evidence that the ecosystem in the riparian habitat is stable and that most of the habitat is being utilized effectively (Voesenek and Bailey-Serres, 2015; Hu et al., 2021; Wang et al., 2022a). However, recent studies show that dam construction affects species diversity and distribution patterns, which results in substantial degradation of riparian habitats (Zheng et al., 2021b; Arif et al., 2022b). The Three Gorges Reservoir (TGR) has a hydrological regime that reverses the natural drought-flooding cycle compared to other reservoirs. In this case, the water level rises in the winter and falls in the summer within the TGR. This mechanism is different from other reservoirs with wide fluctuations (145–175 m mean sea level (m a.s.l.), up to 30 m). Due to the unique hydrological conditions in the TGR region, the plants in this region have a special growth pattern that is continuously submerged in a cycle that repeats each year (Chen et al., 2021; Li J. J. et al., 2021). Consequently, most of the riparian plants could not withstand these huge water level changes and gradually died (Gul et al., 2022; Hu et al., 2022), resulting in severe degradation of the ecological environment in the region and, thus, the functional decline of the riparian ecosystem, which seriously jeopardized the operation of the TGR. Therefore, it is important to know how the riparian plant community responds to dam inundation and to restore vegetation within the hydro-fluctuation zone to improve the area’s ecosystem quality and ecosystem services.

Afforestation is a very successful method for preventing soil degradation, improving the ecological environment, and promoting the restoration of damaged ecosystems (Ren et al., 2017; Behzad et al., 2022; Wang et al., 2022c). Nevertheless, nutrient element balance in hydro-fluctuation zone vegetation restoration has often been ignored. Only a few studies have examined nutrient stoichiometry changes around dams and reservoirs (Kumar and Sharma, 2016; Kumar et al., 2021). And plant growth depends not only on the availability of a single nutrient but also on the balance between various nutrients (Wojciech et al., 2019). As critical elements and basic substances, C, N, and P are indispensable in plant growth (Butler et al., 2017), and their biochemical functions are coupled in plants. Wang et al. (2015) use C:N ratio and C:P ratio as crucial indicators of plant metabolic characteristics and growth conditions, reflecting the ability of photosynthetic carbon fixation and simultaneous absorption of nitrogen and phosphorus. The dynamic balance of plant nutrient requirements and soil nutrients is represented by the N:P ratio (Nikolic et al., 2014). The plant or soil C:N:P stoichiometric ratio in riparian areas can be applied to evaluate riparian health quality (Huang et al., 2019).

Changes between surface and subsurface ecosystems are closely related due to the cycling and feedback of water and mineral nutrients between them (Peichl et al., 2012). C:N:P stoichiometry is a concept that is concerned with the interplay and balance of multiple elements in ecological systems (Ren et al., 2016), and has frequently been used in the study of feedback and interactions between below-ground and above-ground subassemblies of ecosystems (Wang et al., 2022b), especially useful in establishing the connection between soil, plant tissues, and other components of different ecosystems (Zechmeister-Boltenstern et al., 2015; Li J. W. et al., 2019). As per the basic theory of ecological stoichiometry, ecological stoichiometric homeostasis refers to the capability of organisms to keep their own element concentrations and ratios stable in changing environments (Sterner and Elser, 2002; Yu et al., 2011), reflecting the response of biochemical and physiological allocations in organisms to the extrinsic environment (Leal et al., 2017). Stoichiometric homeostasis is reported to be positively associated with vegetation stability and function. It has been verified in herbs (Yu et al., 2011), shrubs (Wang et al., 2016), and trees (Wang et al., 2019). Therefore, vegetation adaptation is associated with stoichiometric homeostasis.

Stoichiometric characteristics of plants result from long-term adaptation to the environment and reflect physiological strategies for optimizing heterogeneous habitats (Zhang et al., 2021). Nutrient allocation patterns and stoichiometry in various plant organs represent the considerations plants confront when accessing above-ground and below-ground resources (Fortunel et al., 2012). Leaves, for example, are primarily responsible for photosynthesis, transpiration, and gas exchange (Rong et al., 2015). Stems are responsible for supporting, storing, and transporting water and nutrients (Fortunel et al., 2012). Roots are responsible for nutrient uptake and support (Li M. et al., 2019), especially fine roots (*d* ≤ 2 mm), which operate as a vibrant contact surface with soil, conduct an important role in nutrient cycling, and are a critical component of soil carbon sequestration (Cao et al., 2020). Soil C:N:P ratios can be used to assess soil nutrient status, control plant growth, and demonstrate plant nutritional conditions (Zheng et al., 2021c). Its relationship with plants is thought to show how plants use resources in an environment that is always changing (Hong et al., 2015). However, many separate studies on the C:N:P stoichiometry of soils (Kumar et al., 2021) or plants (Sardans et al., 2017; Wang et al., 2019) have been conducted, focusing less on the interactions between soils and plants (Kumar and Sharma, 2017). The decision of stoichiometric imbalances in plant-soil systems is unknown (Bell et al., 2014), including how stoichiometric interactions between soils and plants impact ecosystem processes (Kumar and Sharma, 2017) and how environmental factors, successional stages, tree species, and vegetation composition affect the redistribution of nutrients between soils and plants (Ågren and Weih, 2012). Hence, a better understanding of C:N:P stoichiometry in soils and plants will further comprehend sustainable afforestation and nutrient cycling in ecosystems.

This study analyzed the C, N, and P concentrations and stoichiometric ratios of fine roots, stems, leaves, soils, and other soil properties of two flood-tolerant tree species (*Taxodium ascendens* and *Taxodium distichum*). Additionally, the N and P stoichiometric balances were also investigated. As a result, we hypothesized that (i) plants with long-term periodic submergence have the most N and P in their leaves; (ii) deciduous conifer plant species have stoichiometric homeostasis and these vary in organs; and (iii) plant stoichiometric characteristics and soil variables are closely linked.

## Materials and methods

### Study site

**FIGURE 1:**
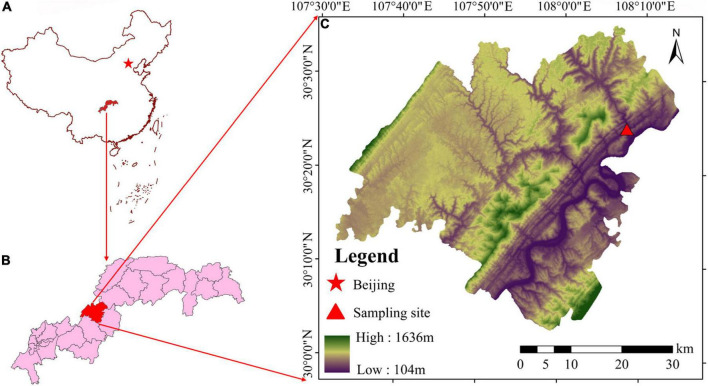
The sample site **(C)** is located in the Three Gorges Reservoir **(B)** of China **(A)**.

The Ruxi River Basin Demonstration Restoration Base in Shibao Township, Zhong County, Chongqing, China (30°24 ′16″∼30°24′56″N, 108°08′03″∼108°08′21″E) was used for this study (Figure 1). The Ruxi River, where the sample plot is situated, belongs to the first-class tributary of the Yangtze River, with four distinct seasons and abundant precipitation (Arif et al., 2022c). The annual average temperature is about 18.2°C and receives about 1,200 mm of annual average precipitation. The frost-free period is 341 days per year with 80% relative humidity. Since the operation of TGR, severe soil erosion and substantial soil heterogeneity have been observed in the reservoir region, and almost all of the original vegetation in this region has been seriously degraded. In order to restore the degraded vegetation, 2 years old *T. ascendens* and *T. distichum* tree seedlings have been cultivated here at 1 × 1 m intervals. The trees were planted at 165–175 m a.s.l. along an important river channel in order to ensure the safety of shipping and to ensure the tolerance limit of trees.

### Sample collection and measurement

Our objective was to explore the plant nutrient stoichiometry at different submergence depths and different sampling times. Three submergence depths and four sampling times were selected: 165 m a.s.l. [Deep Submergence (DS)], 170 m a.s.l. [Moderate Submergence (MS)], and 175 m a.s.l. [Shallow Submergence (SS)] (Chen et al., 2021). The plants were sampled once in July 2018 [during a growth spurt (T1)] and once in September 2018 [just before they were submerged (T2)]. They were then submerged once each in July 2019 [T3] and September 2019 [T4]. A 120 (60 each) healthy and similar *T. ascendens* and *T. distichum* were randomly selected (3 elevations × 4 times × 5 duplicates) to sample the fine roots, stems, and leaves. The collection of fine roots (*d* ≤ 2 mm) was done by using a flat shovel to dig equidistantly in the soil with a radius of 0.25 m around the plant, carefully clean the impurities and soil on the fine roots surface, mix the collected fine roots, and put the quartered part into ziplock bags. The stems and leaves were collected from the middle and upper parts of the plant canopy with branch shears and then mixed and put into zip-locking bags. When collecting soil, remove debris such as dead branches and leaves on the surface, and use a flat shovel to dig out the topsoil (0–20 cm) of corresponding plants. The collected soil samples were mixed evenly, and 500 g of the collected soil samples were taken and brought back with the quartering method.

Rinse the plant samples with tap water to remove impurities from the surface, and then wash the plant samples with deionized water carefully. The cleaned samples were dried for 30 min at 105°C, then at 65°C until the weight was unchanged in an oven and crushed with a ball mill (Lech MM400). Soil samples were naturally dried at room temperature, then ground and screened (1 and 0.25 mm). The C and N contents of all samples were determined by an element analyzer (Elementar Vario EL cube, IDL < 40 ppm). Before the determination, the instrument was calibrated with certified reference materials (CRM) of acetanilide for elemental (GBW06203, certified C content: 71.088%, certified N content: 10.363%), so that the measurement results of the instrument were consistent with the certified value of CRM. The inductively coupled plasma emission spectrometer (ICP-OES, Thermo Fisher ICAP 6300) measures P and K concentrations. Before the determination, CRM of soil (GBW 07404, certified P content: 0.695 g/kg, observed P content: 0.681 g/kg, certified K content: 8.550 g/kg, observed K content: 8.500 g/kg), and CRM of plants (GBW 07605 tea-leaf, certified P content: 2.840 g/kg, observed P content: 2.855 g/kg) were used to verify the elemental analysis methods. A total of 480 C, N, and P samples were tested, respectively. The electrode potential method (Bench Top Professional pH Meter, pH610) measured the soil pH, and the ratio of water to soil was 2.5:1. Using a soil redox potentiometer (HI98120) to measure the soil oxidation-reduction potential (ORP) and the soil water content (SWC) measured by the drying method. The ring knife method determines the soil bulk density (BD).

### Statistical analysis

Using one-way ANOVA to access the differences in C, N, and P concentrations and stoichiometry of soils and various parts of the plants in different submergence groups and different sampling periods with Tukey’s test at the level of *p* < 0.05. Appling the non-metric multidimensional scaling (nMDS) to show how difference changed among different submergence groups using Origin 2022. To determine the links between soil factors and the C, N, and P concentrations and stoichiometry of the plant various parts, the Pearson correlation analysis was applied. To determine the relative impacts of soil factors on plant C, N, and P stoichiometry, a random forest model was used. The model is a statistical learning system capable of producing accurate predictions and explanations. The model was created in R 4.1.2 using the “RandomForest” package.

The equation (*y* = c*x*^1/H^) was used to compute the stoichiometric homeostasis index (1/H) with the R^2^. Before conducting analysis, the data was log_10_ transformed. The form of linear regression models is log_10_*y* = log_10_c + 1/H × log_10_*x*, where *x* is the stoichiometry of soil (resource), and *y* is the stoichiometry of plant tissue. If the model regression relationship was insignificant (*p* > 0.05), 1/H was adjusted to 0, and the plant tissue was classified as “strict homeostasis.” If the model regression relationship was significant (*p* < 0.05), the plant tissue homeostasis degree was categorized as: “strict homeostasis” (0 ≥ 1/H), “homeostasis” (0.25 > 1/H > 0), “weak homeostasis” (0.5 > 1/H > 0.25), “weak plasticity” (0.75 > 1/H > 0.5), and “plasticity” (0.75 < 1/H) (Makino et al., 2003; Persson et al., 2010). Microsoft 2019 software, SPSS 25.0, R 4.1.2, and Origin 2022 were used for statistical analysis, and all graphics were created with Origin 2022.

## Results

### Nutrient concentration variability during flooding and stoichiometric ratios

**FIGURE 2:**
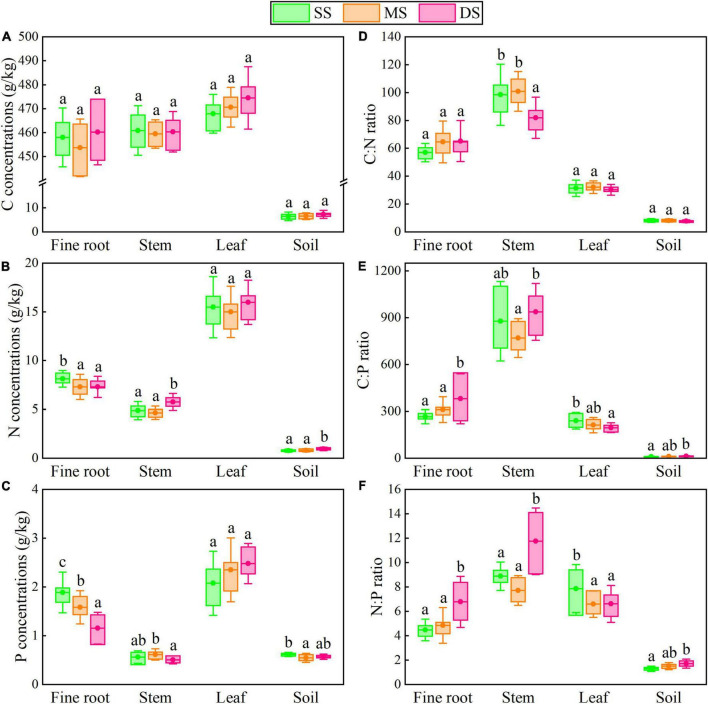
The C, N, and P concentrations **(A–C)** and stoichiometry **(D–F)** in fine roots, stems, leaves, and soils of *T. ascendens* under different submergence treatments. Data shows a significant difference with different lowercase letters (*p* < 0.05) in different submergence treatments (mean with standard error). The boxes give the 25 and 75% percentiles.

**FIGURE 3:**
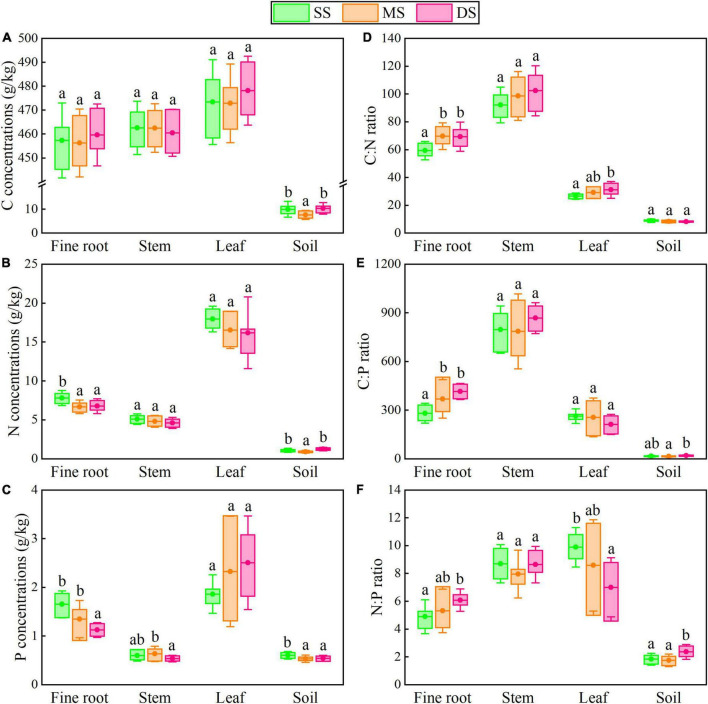
The C, N, and P concentrations **(A–C)** and stoichiometry **(D–F)** in fine roots, stems, leaves, and soils of *T. distichum* under different submergence treatments. Data shows a significant difference with different lowercase letters (*p* < 0.05) in different submergence treatments (mean with standard error). The boxes give the 25 and 75% percentiles.

**FIGURE 4:**
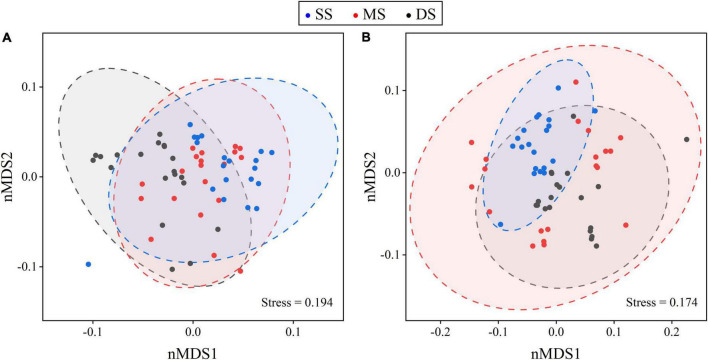
Non-metric multidimensional scaling (nMDS) analysis of plant C, N, and P stoichiometry and soil factors for *Taxodium ascendens* **(A)** and *Taxodium distichum* **(B)** under different submergence treatments. Ellipses show a 95% confidence interval for each submergence treatment.

The mean C:N:P stoichiometric ratios of plants and soils in this study were 323.7:6.6:1 and 14.2:1.7:1, respectively, which had lower C:N:P ratios compared to Chinese plants and soils (Table 1). Specifically, flooding had no significant effect on leaf N and P concentrations or C concentrations in various organs. Still, it significantly decreased the fine root N and P concentrations of *T. ascendens* and *T. distichum* (Figures 2, 3). The C:N ratio was significantly unaffected by the flooding of *T. ascendens* (apart from the stem). The soil and fine root C:P and N:P ratios increased significantly due to flooding while the leaves fell noticeably. Flooding did not affect the C:N, C:P, and N:P ratios of stems, but that of fine roots was noticeably increased. On the contrary, flooding significantly reduced the leaf N:P ratio of *T. distichum* (Figures 3D–F). The soil in SS and DS contained significantly more C and N than the soil in MS, and flooding significantly reduced the soil P concentration of *T. distichum*. The DS had a much greater soil C:P ratio than the MS group (Figure 3E). In comparison, the SS and MS had a much lower soil N:P ratio than the DS (Figure 3F) of *T. distichum*. The results of nMDS analysis also revealed that the C, N, and P stoichiometry along the submergence gradient might be distinct. For *T. ascendens* (Figure 4A), it showed little change under varied submergence treatments. At the same time, flooding greatly impacted *T. distichum* (Figure 4B). As the submergence depth increased, it showed great changes.

**TABLE 1:** Details of plant-soil stoichiometry reported in China.

Study site	C:N:P ratio		References
	Plant	Soil	
Southwestern China	1431:25:1	169:8:1	Zhang et al., 2020
Loess Plateau, China	611:17:1	22:2:1	Cao and Chen, 2017
Subtropical, China	392:18:1	87:13:1	Fan et al., 2015
China’s Forests	1066:11:1	80:4.5:1	Liu et al., 2019
China’s Plant Community	1359:13:1	73:5:1	Zhang J. et al., 2018
China’s Northern Plantation	561.2:8.7:1	54.2:3.5:1	Du et al., 2020
Subtropical Riparian Wetlands, China	177.2:8.9:1	31.8:0.95:1	Yu et al., 2020
China’s Karst Shrubs	638.2:15.8:1	344.3:2.8:1	Zou et al., 2021
Rainy Zone of West China	1538:21:1	74:8:1	Li Z. Y. et al., 2021
Three Gorges Reservoir, China	323.7:6.6:1	14.2:1.7:1	Present study

### Dynamics of plant-soil C, N, and P ecological stoichiometry

**FIGURE 5:**
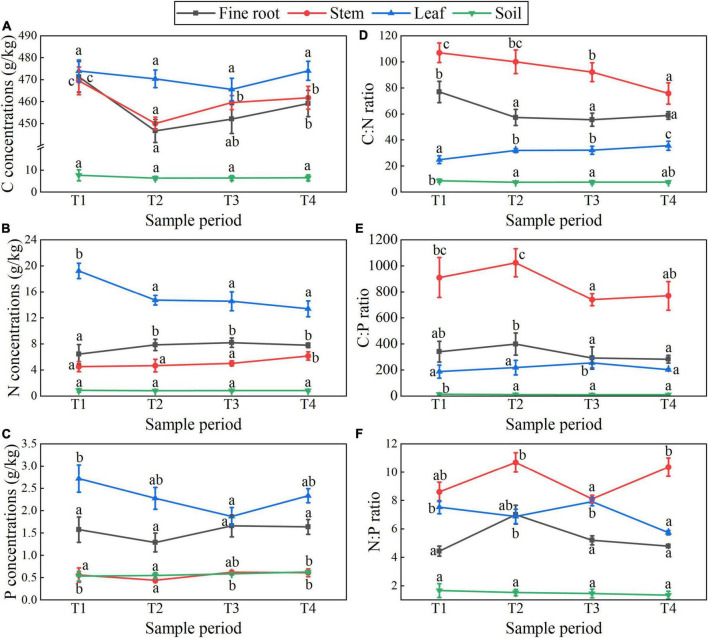
*T. ascendens* showed C, N, and P concentrations **(A–C)** and stoichiometry **(D–F)** dynamics in fine roots, stems, leaves, and soils at different sample periods. Data shows a statistically significant difference with different lowercase letters (*p* < 0.05) across sample periods (mean with standard error).

**FIGURE 6:**
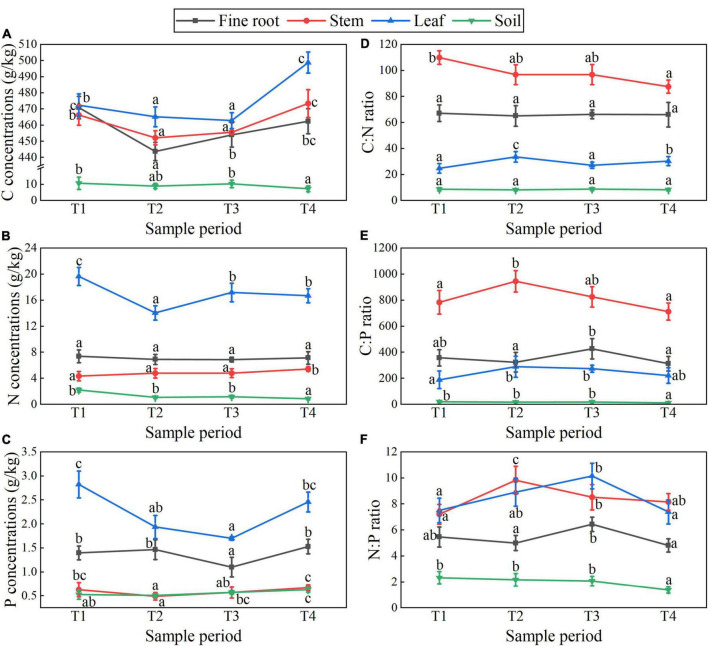
*T. distichum* showed C, N, and P concentrations **(A–C)** and stoichiometry **(D–F)** dynamics in fine roots, stems, leaves, and soils at different sample periods. Data shows a statistically significant difference with different lowercase letters (*p* < 0.05) across sample periods (mean with standard error).

Figures 5, 6 show the plant-soil C, N, and P stoichiometry dynamics of *T. ascendens* and *T. distichum*. The changing trend of C concentration in all tissues of *T. ascendens* and *T. distichum* was parallel and increasing overall. The N:P ratios and N, P concentrations in fine roots and leaves of *T. ascendens* have opposite trends, but they are roughly the same as in *T. distichum*. The fine root P concentration of *T. ascendens* was not noticeably different during the four stages. From T1 to T4, the C:N ratios of fine roots and stems were reduced significantly, while the leaf C:N ratio was significantly increased for *T. ascendens*. The soil N:P ratios, and C, N concentration were not noticeably different in different stages, but soil P concentration increased slightly. In contrast, the soil C:P ratio was reduced imperceptibly by *T. ascendens*. The fine root N concentrations in the four stages did not alter considerably. In contrast, the stem and leaf P concentrations decreased significantly from T1 to T2 and increased from T3 to T4 of *T. distichum*. The stem C:N ratio decreased significantly from T1 to T4, while the fine root C:N ratio had no dramatic difference between the four stages of *T. distichum*. For *T. distichum*, from T1 to T4, the soil C and N concentrations decreased, whereas the soil P concentration increased. The soil C:N ratio did not alter considerably among the four stages, but the soil N:P ratio and C:P ratio decreased. In all *T. ascendens* and *T. distichum* organs, the N:P ratios were less than 14 in each period.

### Stoichiometric homeostasis in plant organs

**FIGURE 7:**
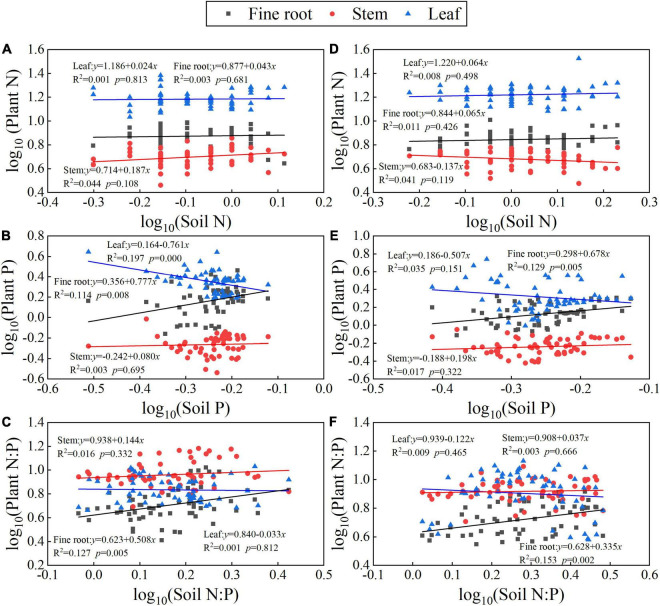
The N, P, and N:P stoichiometric homeostasis coefficients between fine roots, stems, and leaves of *Taxodium ascendens* **(A–C)** and *Taxodium distichum* **(D–F)**.

The N, P, and N:P ratio stoichiometric homeostasis degrees in the fine root, stem, and leaf are shown in Figure 7. For N concentrations, the fine root, stem, and leaves of *T. ascendens* and *T. distichum* were all categorized as “strict homeostasis” (*p* > 0.05) (Figures 7A,D). For P concentrations, the fine root was “plasticity” with 1/H was 0.777 (*p* < 0.05), while the leaf was “strict homeostasis” with 1/H was −0.761 of *T. ascendens* (Figure 7B). The fine root of *T. distichum* was “weak plasticity” with 1/H = 0.678 (Figure 7E). The others were classified as “strict homeostasis.” For N:P ratio, the *T. ascendens* and *T. distichum* fine roots were identified as “weak plasticity” and “weak homeostasis” with 1/H were 0.508 and 0.335 (*p* < 0.05), respectively (Figures 7C,F). The stem and leaves of *T. ascendens* and *T. distichum* were classified as “strictly homeostasis.”

### Relationships among plant organs and soil factors

**FIGURE 8:**
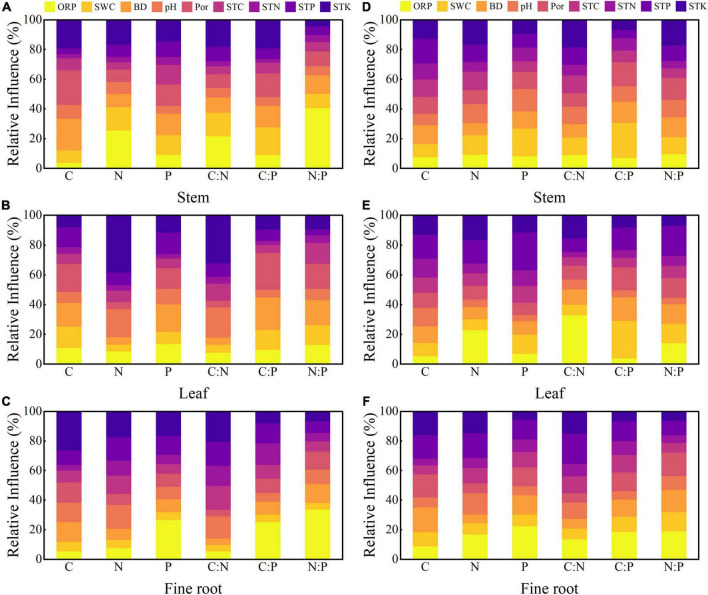
Relative variable importance plots (%) of C, N, and P concentrations and stoichiometry for *Taxodium ascendens* **(A–C)** and *Taxodium distichum* **(D–F)** by random forest models. ORP, soil oxidation-reduction potential; SWC, soil water content; BD, soil bulk density; pH, soil pH value; Por, soil porosity; STC, soil total carbon; STN, soil total nitrogen; STP, soil total phosphorus; STK, soil total kalium.

**FIGURE 9:**
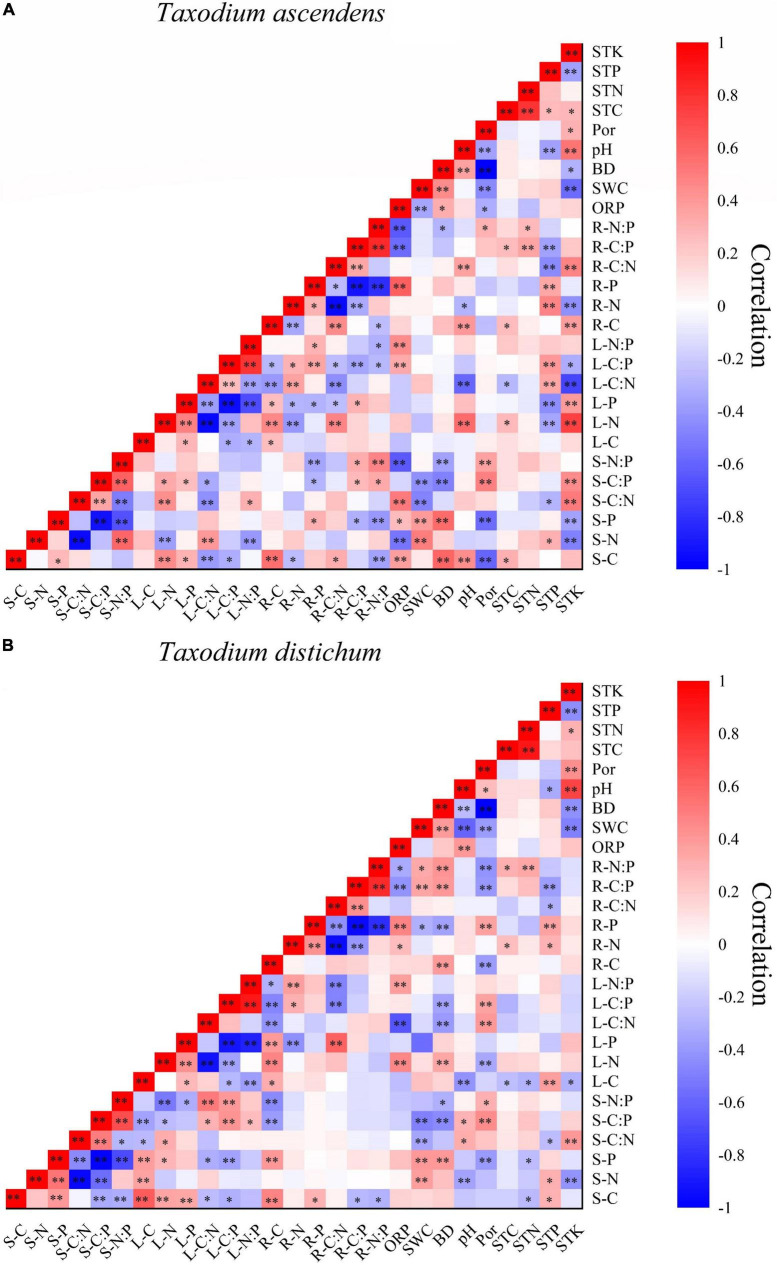
Heat maps of Pearson’s correlation among the C, N, and P concentrations and stoichiometry in various plant parts and soil factors of *T. ascendens* **(A)** and *T. distichum* **(B)**. S-C, stem carbon; S-N, stem nitrogen; S-P, stem phosphorus; S-C:N, stem C:N ratio; S-C:P, stem C:P ratio; S-N:P, stem N:P ratio; L-C, leaf carbon; L-N, leaf nitrogen; L-P, leaf phosphorus; L-C:N, leaf C:N ratio; L-C:P, leaf C:P ratio; L-N:P, leaf N:P ratios; R-C, fine root carbon; R-N, fine root nitrogen; R-P, fine root phosphorus; R-C:N, fine root C:N ratio; R-C:P, fine root C:P ratio; R-C:P, fine root N:P ratio; ORP, soil oxidation-reduction potential; SWC, soil water content; BD, soil bulk density; pH, soil pH value; Por, soil porosity; STC, soil total carbon; STN, soil total nitrogen; STP, soil total phosphorus; STK, soil total kalium. ** Correlation is significant at the 0.01 level (two-tailed); * Correlation is significant at the 0.05 level (two-tailed).

Random forest analysis (Figure 8) and correlation analysis (Figure 9) revealed that soil factors affected the C, N, and P stoichiometry of plant organs. For *T. ascendens* (Figures 8A–C, 9A), STK and soil ORP had the greatest effect on plant organs. Soil BD also greatly impacted the C and P concentrations, and C:P ratio of stems and leaves. For *T. distichum* (Figures 8D–F, 9B). The effects of soil factors on the stem were relatively balanced, and only SWC had a greater impact on the stem C:P ratio. STP had the greatest effect on the *T. distichum* leaves, followed by soil ORP, which had a greater impact on the leaf C:N ratio. SWC also had a higher impact on leaf C:P ratio. Soil ORP and STP had the greatest effect on *T. distichum* fine roots.

The correlation coefficients among plant C, N, and P stoichiometry and soil factors ranged from small to large and included both positive and negative coefficients (Figure 9). The N concentration was dramatically positively connected with the P concentration in all plant tissues (except the stem of *T. ascendens*). The C concentration was notably positively connected with P concentration in the stems and leaves of *T. ascendens* and *T. distichum*. STP was notably positively connected with the fine root N and P concentrations (*p* < 0.05). The SWC was extremely positively connected with the stem N and P concentrations (*p* < 0.01). Leaf N concentrations were extremely positively connected with fine root C (*p* < 0.01). Fine root P concentrations and leaf P concentrations were significantly negatively associated (*p* < 0.05) with *T. ascendens*. Leaf P concentrations and fine root N concentrations were notably negatively connected (*p* < 0.05). Stem C and P and leaf C, N, and P were extremely positively connected with the fine root C (*p* < 0.01) of *T. distichum*. Furthermore, for *T. ascendens* (Figure 9A), stem C was notably positively connected with fine root C, stem P, leaf N and P, ORP, BD, pH, and STC (*p* < 0.05). For *T. distichum* (Figure 9B), ORP was extremely positively connected with leaf N and fine root P. And pH was extremely negatively connected with stem N and leaf C (*p* < 0.01).

## Discussion

### Nutrient concentration patterns and stoichiometric ratios

Plant genetic traits and resource acquisition strategies play decisive roles in influencing plant nutrient dynamics (Horodecki and Jagodziński, 2017). The variation of C, N, and P stoichiometry of *T. ascendens* and *T. distichum* indicated that the plant organs were affected by the corresponding tissue structure and functional differentiation, resulting in different requirements for nutrient elements (Sardans et al., 2017). The C in fine roots, stems, and leaves was unaffected by flooding significantly (Figures 2A, 3A), and the ratio was about 1:1:1, showing that C allocation in all organs was largely balanced. Furthermore, the C concentration of each organ was much higher than the corresponding surface soil, indicating that the C concentration and C sequestration capacity of the two tree species are higher. The atmosphere was the main source of C element in plants, so soil C element have relatively little effect on it. Changes in plant tissue stoichiometry may occur during plant growth (Gao et al., 2019). The C, N, and P stoichiometries in each organ were considerably altered throughout the plant growth stage gradient. In all four stages, the C concentration in each organ of *T. ascendens* and *T. distichum* demonstrated a consistent trend, indicating C element integrity in metabolism. It also shows that plant C has not participated in plant production directly. Instead, it primarily serves as a relatively stable plant skeleton (Pang et al., 2021).

Leaves had the highest concentrations of N and P of the two tree species (Figures 2B,C, 3B,C), indicating the plant organ metabolic function is compatible with the N and P allocation (Shi et al., 2021; Yin et al., 2021). And flooding did not have a significant effect on it, mainly because tree leaves require more substances rich in N and P (such as nucleic acids, enzymes, and transporters) to engage in metabolism (Zhang B. et al., 2018). The plant economics spectrum hypothesis indicates co-variation between fine root traits and leaf traits to optimize a whole-plant ecological strategy (Valverde-Barrantes et al., 2017). This phenomenon also indicates that under flooding stress, N, P, and other nutrients absorbed by plants are more likely to be allocated to leaves to complete normal physiological activities. It may be an ecological strategy of tree species to adapt to flooding stress. This finding proves our first hypothesis. Stems and fine roots are the main organs for absorbing and storing N and P. They also serve as support and fixation. Thus, more C was required to build a body skeleton (Yin et al., 2021). Flooding dramatically increased fine root C:N, C:P, and N:P ratios (Figures 2B–F, 3B–F), indicating the N and P utilization efficiency of fine roots was improved under flooding (Cao et al., 2022). The nMDS analysis (Figure 4) showed that the changes in the C, N, and P stoichiometric characteristics of *T. distichum* were larger than those of *T. ascendens* along the intensity of flooding increases, which indicated that the stability of *T. ascendens* was stronger under flooded conditions.

Plants can live by altering their stoichiometry. The relationships of C:N:P ratios among plant tissues reflect biochemical tradeoffs in plant growth strategies and nutrient acquisition (Luo et al., 2021). As essential physiological indicators of plants, C:N and C:P ratios can reflect the defense and competition strategies of plant species (Wang et al., 2015). In addition, they can also illustrate the plant N and P usage efficiency under the same carbon fixation capacities (Zhang B. et al., 2018). Fine roots and stems have greater C:N and C:P ratios, implying these organs invest a lot of C in supporting functions (Yin et al., 2021). The N:P ratios in all organs of the two tree species in the current study were below 14, and a tendency toward N limitation was shown in their development (Tessier and Raynal, 2003). The most probable cause seems to be anthropogenic eutrophication. Soils near rivers have higher P concentrations than undisturbed habitats due to the human effects of substances such as agricultural runoff, domestic sewage discharge, and detergents containing P. Thus, the hydro-fluctuation zone ecosystems can be transformed into potentially N-limited ecosystems from P-limited ones by anthropogenic eutrophication (Yan et al., 2016).

Soil C and N concentrations fell somewhat as time passed, whereas soil P concentrations rose (Figures 5A–C, 6A–C). The reason is that flooding reduces soil porosity, oxygen content, and ORP, which in turn reduces the physicochemical activity of soil. These alterations will further stifle the transformation and decay of soil litter and organic material, reduce soil organic carbon content, and affect the soil N cycle (Yang et al., 2022). Due to poor soil oxygen and low porosity, N mineralization was reduced while denitrification was increased (Fellows et al., 2011), promoting soil N loss and reduction. Meanwhile, plants can absorb mineral N from the soil and lower the N concentrations in the hydro-fluctuation zone, reducing the soil N concentrations to improve water quality (Ye et al., 2012). Soil P derives mainly from inherent soil components and is easily sedimented, reducing P element availability (Lambers et al., 2015). The hydro-fluctuation zone can also absorb a large amount of available P from surface runoff during exposure (Shu et al., 2017; Chen et al., 2022). Long-term anaerobic or frequent flood events lead to further deposition of soil P, thus increasing soil P concentrations. The soil C:N ratio may also be used as a stoichiometric index for characterizing the rate of soil organic matter mineralization and available N release and reflects the plant’s net primary productivity (Marty et al., 2017). Because C and N are structural components with relatively constant consumption and accumulation ratios (Marty et al., 2017; Pang et al., 2021), the C and N accumulation ratios are synchronous, so the soil C:N ratio changes little at different stages. Soil P concentration was the primary determinant of soil C:P and N:P (Tian et al., 2010), and the reduction in C:P and N:P was caused by increasing P concentration.

### Stoichiometric homeostasis of plant tissues

The plant’s ecological stoichiometric homeostasis is closely related to its ecological strategies and adaptations. The stoichiometric balance degree appears to be different in different tissues of the same plant, reflecting the basic balance of nutrient input and distribution (Wang et al., 2016). The stoichiometric homeostasis of *T. ascendens* and *T. distichum* is relatively strong (Figure 7), indicating that the two tree species have gradually formed strong adaptability during the long-term flooding stress process. The stems and leaves of these plants are devoted to maintaining homeostasis (Su and Shangguan, 2021). Plant growth and biomass accumulation are dependent on the leaves, which are the main photosynthetic organs. Therefore, to guarantee optimal biophysical features, nutrients are managed at a specific level (Wang et al., 2018). In addition to transporting and storing, the stems maintain the N and P homeostasis, which aids in the coordination of other organs. Therefore, environmental fluctuations were minimized by these species’ resource use and storage capabilities, and our second hypothesis is partially validated. Only the fine roots of the P element and the N:P ratio showed plastic in the current study. The main reason was that fine roots were organs that contacted and exchanged nutrients with the soil, so the fine roots of plants could quickly sense changes in the soil environment. Thus, they strongly correlate with environmental variables, contributing to leaf nutrient homeostasis (Yin et al., 2021). The other reason might be associated with the fact that the fine roots are far away from the assimilation organs and the short life span and continuous renewal of fine roots. In metabolism, the P element is crucial for cell division, leading to the change or mobility of the P element in fine roots, resulting in plastic (Fu et al., 2020). Moreover, high P concentrations and availability can impede the synthesis of P-containing substances (Pii et al., 2015). Such consequences will alter plant physiological processes (i.e., N-fixation, photosynthesis) (Ren et al., 2016), ultimately resulting in changes in plant growth.

The N:P stoichiometric homeostasis was superior to N or P alone in terms of assessing the plant homeostasis state. Stoichiometric homeostasis describes how organisms consume resources and store them throughout development (Yu et al., 2010; Blouin et al., 2012). Furthermore, the N:P ratio can disclose the P availability in the ecosystems and bring to light nutrient re-translocation between soils and plants (Fan et al., 2015; Cao and Chen, 2017). It also implies that the P element has the greatest impact on the N:P ratio (Luo et al., 2021). Thence, the N:P ratio may better reflect the strong intrinsic link between soils and plants’ nutrients (Zhang et al., 2019). This may also be a growth strategy for *T. ascendens* and *T. distichum* to accommodate the flooded environment. The two plant species use their underground root systems to get nutrients, store them, and move them. This allows them to keep a high level of homeostasis for their above-ground parts (stems and leaves) to withstand flooding, making them dominant in changing environments and suitable for the hydro-fluctuation zone of the TGR area. Furthermore, because of the high metabolic activity of fine roots, considerable quantities of N and P are needed to synthesize carrier enzymes that actively absorb nutrients from soil solutions (Garrish et al., 2010). Hence, roots may be a more reliable indication of soil nutrient conditions (Schreeg et al., 2014). The second hypothesis has so far been fully verified.

### Relationships of C, N, and P concentration and stoichiometry among organs and their responses to soil factors

Long-term and frequent inundation and de-inundation events lead to changes in soil nutrient concentration, texture, porosity, aggregation stability, pH, and ORP (Baastrup-Spohr et al., 2016; Shu et al., 2017). Soil pH and ORP are major regulators of soil nutrient solubility and availability (Schramm et al., 2009; Asmamaw et al., 2022). Many variables, including plant functional groups and soil nutritional conditions, can affect nutrient stoichiometry and distribution across plant organs (He and Dijkstra, 2014; Sardans et al., 2017). Our research demonstrated that the plant’s C, N, and P stoichiometry strongly relates to soil properties. The C, N, and P concentrations in fine roots were extremely connected with that in soil, and that in above-ground parts (leaves and stems) were extremely connected with fine roots. These findings suggest that the soil nutrients regulate the nutrient ecological stoichiometry in above-ground parts through the root system (Zhou et al., 2018). For *T. ascendens*, except for leaf P, the N and P in fine roots, stems, and leaves were not connected with leaf C, suggesting that C assimilation differs from N and P fixation (He et al., 2015). Leaf C, however, was substantially positively associated with stem N, stem P, and leaf P of *T. distichum*, indicating that they were synchronized.

Among soil elements, the P element is most closely related to plants. The findings show that soil nutrients, particularly P elements, are the primary drivers of those plant elements (Yuan et al., 2011). There was a more significant P limitation in the two tree species. The reason may be that they were beginning their exuberant plant growth stage. Therefore, substantial amounts of phosphorus were required to maintain the high primary productivity required for plant development, thereby intensifying the P limitation (Qi et al., 2022). Soil K is also closely related to plant nutrient changes, as soil K release depends mainly on physical adsorption to clay particles. In the hydro-fluctuation zone, flooding may have accelerated the erosion of fine particles deposited and leaching of soil available K, resulting in a reduction in the soil available K (Ye et al., 2019), while an increase in K adsorption after soil drainage (Venterink et al., 2002). Therefore, frequent flooding and drying lead to changes in soil K content, which affects nutrient dynamics in plants. Soil pH is a comprehensive indicator of nutrient availability in the soil that can adjust to various biological activities (Pang et al., 2021). Our results revealed that N:P ratios were closely connected to P concentrations in all plant tissues, indicating that the shift in plant N:P ratios was primarily caused by the change in plant P. Furthermore, soil P was a major determinant of P levels and N:P ratios in various plant organs (Liu et al., 2019), suggesting that plant N:P ratios may be affected by soil P. The significant positive association between SWC and stem N and P indicates that stems are crucial in storing water and N and P. As a result, our third hypothesis was also well supported. The interaction of N and P revealed a significant positive connection in each organ, and this closely connected relationship exhibited strong coordination to keep the plant functioning in severe environments (Yin et al., 2021). Meanwhile, N and P are the fundamental elements that jointly collaborate on various physiological processes. Therefore, under the same environmental conditions, the change of N and P is consistent (Huang et al., 2019).

## Conclusion

The current study examines the nutrient stoichiometry variation law in multiple organs of flood-tolerant plants and relates it to the response of these plants to flooding and soil phenomena in hydro-fluctuation zones. Our results indicate that flooding affected the covariance of each organ, which led to different influences of water level changes on the concentration of the same element in each organ. Under the presumption that the plants are not deficient in nutrients, the two tree species can adapt positively to fluctuations in water level and maintain stable ecological stoichiometry. The plants can effectively balance the element concentration ratio in each organ and adapt to flooding by balancing the C element in each organ and retaining high N and P levels in their leaves. Their N:P ratios were less than the critical ratio of 14, suggesting that plants growing here may be experiencing N-limitation after years of frequent flooding. The stoichiometric characteristics of C, N, and P in various organs of *T. ascenden*s and *T. distichum* were affected by soil factors. The fine roots were the most susceptible to soil factors. As a result, fine roots are capable of absorbing and transporting nutrients. This maintains the homeostasis of above-ground parts, which allows the plants to adapt to flooding stress. The study also found that the soil may be at risk of C and N loss after repeated flooding. In addition, human activities may cause P eutrophication in the soil and water. Our study concluded that tree growth is directly related to ecological stoichiometry, which can help us better understand plant-environment relationships. It has substantial implications for preserving and restoring vegetation in the hydro-fluctuation zone of the TGR.

## Data availability statement

The original contributions presented in this study are included in the article/supplementary material, further inquiries can be directed to the corresponding author.

## Author contributions

DD and MA conceptualized this study. DD, MA, ML, JL, XH, QG, FY, and CL designed the experiments and analyzed the data. DD, ML, JL, XH, QG, and FY were responsible for sample collections. DD, MA, and CL wrote and revised the manuscript. MA and CL supervised the research. All authors contributed to the article and approved the submitted version.
